# State of the Patients in the Bristol Dispensary, from the 1st of March to the 1st of April, 1801

**Published:** 1801-05

**Authors:** 


*State of the Patients in the Briſtol Diſpenſary, from the 1ſt of March to the 1ſt of April,* 1801.

| | | | |
|---|---|---|---|
| Typhus Fever - - - - | 44 | Conſumptions - - - - | 8 |
| Stomach Complaints - - | 8 | Podagra - - - - - - | 1 |
| Continued Fever - - - | 17 | Hæmiplegia - - - - - | 1 |
| Aſthma - - - - - - - | 2 | Scorbutic, &c. - - - | 3 |
| Peripneumonia - - - | 5 | Tabes Dorſalis - - - | 1 |
| Dropſy - - - - - - | 9 | Pleuritic - - - - - | 2 |
| Worms - - - - - - - | 3 | Aſthenia - - - - - - | 1 |
| Cough and Dyſpnœa - - | 3 | Convulſio - - - - - - | 1 |
| Rheumatiſm - - - - - | 5 | Hæmoptoe - - - - - - | 2 |
| Ophthalmia - - - - - - | 1 | Uterine Hæmorrhage - - | 2 |
| Bowel Complaint - - - | 7 | Hyſteria - - - - - - | 1 |
| Puerperal Fever - - - | 1 | | |

